# Reducing traffic violations in the online food delivery industry—A case study in Xi'an City, China

**DOI:** 10.3389/fpubh.2022.974488

**Published:** 2022-10-06

**Authors:** Xin-wei Lu, Xiao-lu Guo, Jing-xiao Zhang, Xiao-bing Li, Li Li, Steven Jones

**Affiliations:** ^1^College of Transportation Engineering, Chang'an University, Xi'an, China; ^2^Hebei Zhangjiakou Municipal Development and Construction Holding Group, Zhangjiakou, China; ^3^School of Economics and Management, Chang'an University, Xi'an, China; ^4^Center for Urban Transportation Research, University of South Florida, Tampa, FL, United States; ^5^Civil, Construction and Environmental Engineering, The University of Alabama, Tuscaloosa, AL, United States

**Keywords:** online food delivery, hierarchical food delivery framework, traffic violations, crashes, demand shunting, conditional PSM model

## Abstract

Online food delivery (OFD) is one of the top industries in the Online-to-offline (O2O) commerce sector. Deliverymen need to complete a large number of delivery orders in limited default time every day, which cause high working stress to them. Therefore, a high level of traffic violations and crashes by deliverymen and corresponding negative impact on public safety are observed. To reduce traffic violations by deliverymen and resulting crashes, a hierarchical online food delivery framework is proposed, which is based on data from questionnaire surveys conducted in Xi'an City, China. The study includes the analysis of the root cause correlated with traffic violations during online food delivery as part of an empirical study on the priority delivery fee by applying a conditional price sensitivity measurement (PSM) model. The feasibility and rationality of the framework are further investigated by using cross analysis of urban dwellers' occupation, income, and commuting cost. The results identify that, through rationally shunting the demand of online food delivery, prolonging the default delivery duration, and providing diversified delivery services, the proposed hierarchical online food delivery mechanism is able to relieve the stress of deliverymen during peak hours of food requests. This reduces the willingness of deliverymen to engage in traffic violations, and other risky behaviors during food delivery trips. All of which facilitate high-quality and timely online food delivery service while enabling improved safety of deliverymen and others as part of enhanced public safety and health.

## Introduction

With the rapid development of “Internet+” and smart phones, the online-to-offline (O2O) business model has been able to expand rapidly in the business world. This has resulted in people being able to have more convenient access to bookings of many consumer and transport related services such as rental cars, tours, hotels, flights, train tickets, and local cuisine. Owing to the rapid growth of the O2O business model, online food delivery is exploding as one of the top industries in the O2O commerce sector (1–4). This industry is growing fast in many counties, such as China, United States, India, and Italy (5–9). Particularly, in China, this industry has exploded (10–12). Indeed, according to recent statistics, the number of online food consumers in China has reached 0.469 billion by mid 2021, and the scale of the online food delivery market of China was ~835.2 billion yuan by the end of year 2020 (13). The rapid growth in consumer demand for online food has spawned a large-scale group of deliverymen within the food delivery system. Statistics show that merely on the Meituan service platform alone, there were about 2.95 million deliverymen in 2020 (14).

However, as the fast development of the OFD market, deliverymen feel great working pressure, such as the large number of orders to delivery in a day, limited default delivery time, the seeking of more salary, and the peer competition.

In China, electric scooters (including motorcycles, named as e-scooters) are the main travel mode used by the deliverymen. Compared to conventional passenger vehicles, e-scooters are much more affordable, and the small size of e-scooters enables deliverymen to take flexible routes in complex and congested city traffic environments (see details in Appendix 1).

However, deliverymen conduct lots of repeated and unstoppable traffic violations by taking the advantage of the benefits and convenience of using e-scooters, including but not limited to reverse-lane driving, red-light running, speeding, disregard of roadway signs and markings, and non-yielding to pedestrians. Actually, traffic violation is regarded by many deliverymen as an illegal but practical way to solute the working pressure partly. They can complete more orders, avoid delivery timeout and win the peer competition by conducting traffic violations during the delivery (information obtained *via* in-depth interviews with deliverymen by authors). Thus, rapid increase of traffic crashes and violations in online food delivery field are observed more and more frequently in recent years. For example, in the first half of 2021, traffic police in Jinan, China investigated and dealt with 4,304 deliverymen traffic violations (15). In November 2020, more than 800 deliverymen traffic violations were observed in Xi'an, China (16). In this regard, various measures to address this matter have been introduced throughout China. For example, Shanghai has implemented a “*one deliveryman, one electric motor car, one license and one code*” mechanism since 2018 (17). Xi'an traffic police have worked with fast food and takeout enterprises to establish a mechanism that also guarantees that deliverymen that are found to violate traffic laws will be dismissed and subsequently blacklisted for such work (18).

Although the above countermeasures are effective in reducing traffic violations by deliverymen in the short term to a certain extent through strengthening online food industry regulations, increasing penalties and encouraging societal supervision. However, due to the limited resources of traffic police, superficial enterprise supervision, and limited societal supervision, the effectiveness of these countermeasures is limited in the long term. Therefore, the level of such traffic violations has not been radically reduced, and it continues to be difficult to achieve a win-win goal of meeting the demand for “fast” food delivery and reducing the level traffic violations by deliverymen.

Therefore, this study develops a new hierarchical online food delivery mechanism by quantitatively analyzing the survey data collected from citizens living in Xi'an, China. The paper study to evaluate the root reason for online food delivery traffic violations, and propose empirically derived solutions to improve both the safety and effectiveness of online food delivery services.

## Literature review

### O2O business development in the online food delivery industry

The online food delivery (OFD) industry is one of the top growing industry in the O2O business and has received lots of attention from researchers in recent years (19–23). Currently, research on this area is mainly focused on the following four aspects:

(1) Customer satisfaction and loyalty analysis. Customer satisfaction and loyalty is important since it is directly related to the survival and development of the business. Thus, scholars have paid lots of attention to research in this area. For example, Annaraud and Berezina (24) revealed a strong positive impact of customer satisfaction on behavioral intentions to use OFD service in the U.S. market. Food quality, past online experience, and service fulfillment was found to affect customer satisfaction in OFD services. Gunden et al. (25) examine consumer's intension to use OFDS by using comprehensive structural model and data collected from 605 US respondents. The results show that performance expectancy is the strongest predictor of intensions to use OFDS, followed by congruity with self-image. Yeo et al. (26) examine the structural relationship between convenience motivation, post-usage usefulness, hedonic motivation, price saving orientation, time saving orientation, prior online purchase experience, consumer attitude and behavioral intention toward OFD services. The study proposes an integrative theoretical research model based on the Contingency Framework and Extended Model of IT Continuance. The results imply that the proposed hypotheses were supported, except for the relationship between prior online purchase experience and post-usage usefulness. Suhartanto et al. (27) identified that food and e-service quality have both direct and indirect effects on online customer loyalty, respectively. Whereas, Chandrasekhar et al. (28) analyzed the consumer perception for several big OFD companies (e.g., Zomato, Swiggy, Food panda) in India and found none of the companies was given the highest rank in terms of food delivery service quality. Devipriya et al. (29) identified that on-time food delivery is critical for a good relationship between consumers and the service platforms (e.g., Just Eat, Grubhub, Uber Eats, and Dominos).

(2) Delivery route optimization and service quality improvement has been investigated by various researchers. For example, Soto et al. (30) carried out different optimization experiments based on Google maps to minimize delivery costs and maximize the number of food deliveries with the best delivery route by using a bioinspired algorithm (PSO). Liao et al. (31) proposed a multi-objective scheduling model to deal with the green food delivery routing problem based on a two-stage solution strategy with Tabu Search (TS) and Genetic Algorithm (GA). Liu et al. (32) developed a deep inverse reinforcement learning (IRL) algorithm to generate accurate navigation routes based on historical GPS trajectories even when road network information is unknown. Dai et al. (33) provides a systematic method for the O2O platforms to optimize order assignment and routing with considering different types of drivers, such as in-house drivers, full-time drivers and part-time ones. Li et al. (34) studied the routing optimization model and algorithm of delivery route. A time penalty cost is introduced first. Then, the objective function is set as the sum of the incremental costs of takeout distribution. Then k-means and genetic algorithms are used to identify the optimal routes.

(3) Food quality and public health. Tang et al. (35) have put forward a food quality and safety risk assessment system by utilizing the Delphi method. Stephen et al. (8) discussed the relationship between the food delivery app and the level of overweight people and obesity in America.

(4) Future trends, platform polices and legal issues. Luo et al. (36) proposed that future delivery services will be mainly completed by automated vehicles (AVs) since AVs can help reduce traffic crashes, injuries and are more environmentally friendly. In this case, an algorithm was proposed to solve the Delivery Reward Maximization (DRM) problem with two special cases as a linear and two-dimensional DRM. Whereas, Liu (37) developed a mixed integer programming (MIP) model for online fleet dispatch operations with lightweight drones by allowing dynamic order information, arbitrary pickup and delivery locations. Further, Sun (12) explored the labor conditions of three Chinese food delivery platforms, namely Baidu Deliveries, Eleme, and Meituan, including salaries, classifications, and platform management.

Unfortunately, research gaps still exist despite the studies mention above. First, few studies have specifically focused on traffic violation issues by deliverymen. Indeed, such a phenomenon has not received enough attention and corresponding in-depth study. Second, merchants and platforms are currently committed to continuously improving back-end algorithms, optimizing order allocation to improving delivery speed and customers' satisfaction. Traffic violation reduction is not considered. But the space for future such route optimization and order allocation is pretty limited since usually global optimum does not exist for sure. Third, the manual delivery is and will be the main delivery choice in the near future. Delivery by automated vehicles and drowns is still in experimental stage, which needs more test and formulating corresponding laws and regulations. Therefore, the traffic violation issues by deliverymen deserves more and in-depth study.

### Traffic violations research

As mentioned previously, deliverymen in China mainly use e-scooters or motorcycles as the main vehicular mode, and therefore it is useful to review appropriate research on motorcycle traffic violations.

Current studies agree that traffic safety risk for motorcycles is much higher than that for passenger vehicles (such as cars), thus safety research into motorcycles has received much attention (38, 39). Key research topics include: (1) Driving behaviors that contribute to more than 80% of the crashes. Based on 104 collected articles, Yousif et al. (40) found that driving speed, visibility and fatigue from driving are the three key factors leading to motorcycle crashes. Whereas, Goh et al. (39) confirmed the positive relationship between risky driving behaviors and crash outcomes; (2) Safety equipment and effectiveness (i.e., helmets and reflective clothing). Helmets have been found to be effective in reducing head injuries for both adults and children (38, 41). However, helmet wearing rates are still low in developing countries (such as Cambodia and Jamaica) (41, 42); (3) Surrounding environmental factors. Previous studies have revealed that environmental factors, such as weather and roadway conditions (i.e., curve segments), and lighting are significantly correlated with the crash severity (43–45). Besides these, mobile phone use (i.e., calls, texting) was also found to have a significant negative impact on traffic crashes and associated injury severity (46, 47).

Although there are some related researches on traffic violations by conventional motorcycle drivers, limited insights can be applied from these more general traffic safety studies to the case of deliverymen. This is because of the distinct trip purposes and motivations of deliverymen. Furthermore, deliverymen often wear helmets and uniform reflective clothing, do not drive-under-influence, and are mostly young and middle-aged adults. Additionally, OFD usually avoid morning traffic peak hours. Thus, any conventional safety countermeasures toward motorcycles are not necessarily appropriate for deliverymen. Consequently, the motivations behind traffic violations by deliverymen needs to be evaluated and analyzed to eliminate the corresponding illegal riding behaviors.

### Working pressure and mental health research

Working pressure in OFD industry is regarded as medium above compared with other industries (48). Most deliverymen can feel the stress during work (49, 50). Jiang (51) studies the work pressure dilemma caused by the practice of distribution through social labor space in the online delivery industry first. Then he analysis and confirm the use of comprehensive intervention methods of social work can alleviate the pressure of the delivery staff group. Zhang (49) analyzed the pressure resources by using deliverymen working in Guangzhou area as an example and found that the reducing of delivery time, complex delivery environment, customers' expectation, and peer competition are the main sources of working pressure. Li (52) stands on the position of deliveryman group, analyzed and excavated the plight of deliverymen under the background of artificial intelligence through data mining method, compared it with the focus of government policies and gave suggestions from the perspective of platform, government and consumers. Long (53) studied the mental health of deliveryman, including their mental health, perceived social support, mental capital, and psychological and burnout capital. She concluded that delivery staff's mental health is good. Perceived social support, job burnout, psychological capital and mental health were positive correlated. Liu et al. (54) studied the hidden dangers of deliveryman group, including dangers from traffic, platform algorithm, reward and punishment mechanism. Then, he made suggestions to government, platform and outsourcing company, respectively.

Almost in all of these papers, working pressure from delivery dimension has been recognized and confirmed. The countermeasures and solutions mainly focus on psychological counseling, algorithm optimization and consumers' forgiveness. It's a pity all these existing countermeasures still cannot solute the working pressure thoroughly. Therefore, new countermeasures should be studied and raised, especially focus on the customer end, which is the source of demand. The number and distribution of demand determines the number of delivery orders and delivery time to a large extent, which need more and in-depth research.

This article explores safety strategies to reduce deliverymen traffic violations in the OFD industry in China. The study takes people from Xi'an as the research object to conduct both online and offline surveys. Root cause of deliverymen traffic violations is analyzed based on the survey data. Then, this study also develops a new hierarchical online food delivery mechanism where merchants and delivery platforms can set up a variety of delivery services. Each level of delivery service corresponds to different delivery times and charges; thus customers are encouraged to select desired delivery level to meet their own dining needs. In this way, the peak-hour food ordering demand are further flattened, and deliverymen are less pressured with extended delivery time. Most importantly, deliverymen are less willing to conduct traffic violations.

## Methods

To better assist the development of safety countermeasures for traffic violations caused by deliverymen, root cause analysis of such traffic violations is of key importance by using Xi'an as the research object. Xi'an is one of the new first-tier cities and national central cities in China. Also, it is a popular domestic and international tourism destination. The GDP of Xi'an was 1068.8 billion of year 2021 (55). The population was 12.96 million by the end of year 2020 and the catering income was 35,277 million Yuan in year 2020 with a rapidly booming OFD industry (56). The OFD market is large enough to be a typical representative of other provincial capital and first/second-tier cities. Meanwhile, similar to other cities in China, the fundamental issue of traffic violations by deliverymen is a pressing need that needs to be addressed. Only through a deeper understanding of the root cause of deliveryman traffic violations can we develop an effective strategy to solve the problem.

### Questionnaire distribution, revoke, and inspection

This empirical study designed and distributed two questionnaires for collecting primary research data. The first questionnaire was issued to consumers regarding 'The Quality of Takeout Delivery and Traffic Safety Status in Xi'an City' (noted as Questionnaire #1). This questionnaire took online food consumers in Xi'an as the survey object and applied the method of combining the two-stage sampling method with the random survey method. A total of 1,225 questionnaires were collected, wherein 1,061 valid questionnaires were included with an effective rate of 86.61%. Distribution of their occupations include student (42.86%), company employee (18.80%), public institution staff (15.32%), merchant (4.70%), Staff in service industry (3.48%), retiree/unemployed (2.35%), freelance (2.07%), worker (1.69%) and else (8.73%). Note: Staff in service industry includes waiter, driver, salesman, customer service staff. Freelance includes writer, artist, photographer, tourism guide, network anchor, etc. Public institution staff includes the persons work in university, research institution, government and so on.

The second questionnaire was issued to deliverymen concerning 'Traffic Safety Status of Deliverymen in Xi'an City' (noted as Questionnaire #2). Additionally, in-depth interviews have been conducted with 23 takeout deliverymen. This questionnaire took the deliverymen whose occupation was OFD within a year in Xi'an as the survey object and applied the random survey method. A total of 232 questionnaires were collected, wherein 203 valid responses were included with an effective rate of 87.5%. Among them, females accounted for 2% and the age expanded from 20- to 40 years old.

The test results of the reliability and validity of two questionnaires were good (see Table 1).

**Table 1:** Summary of test results for reliability and validity of the questionnaire.

**Tests**	**Reliability test**		**Validity test**	
	**Cronbach's Alpha**	**Result**	**KMO**	**Significance**
Questionnaire #1	0.755	Good	0.692	0.000
Questionnaire #2	0.775	Very good	0.643	0.000

### Root causes of deliveryman traffic violations

**Figure 1:**
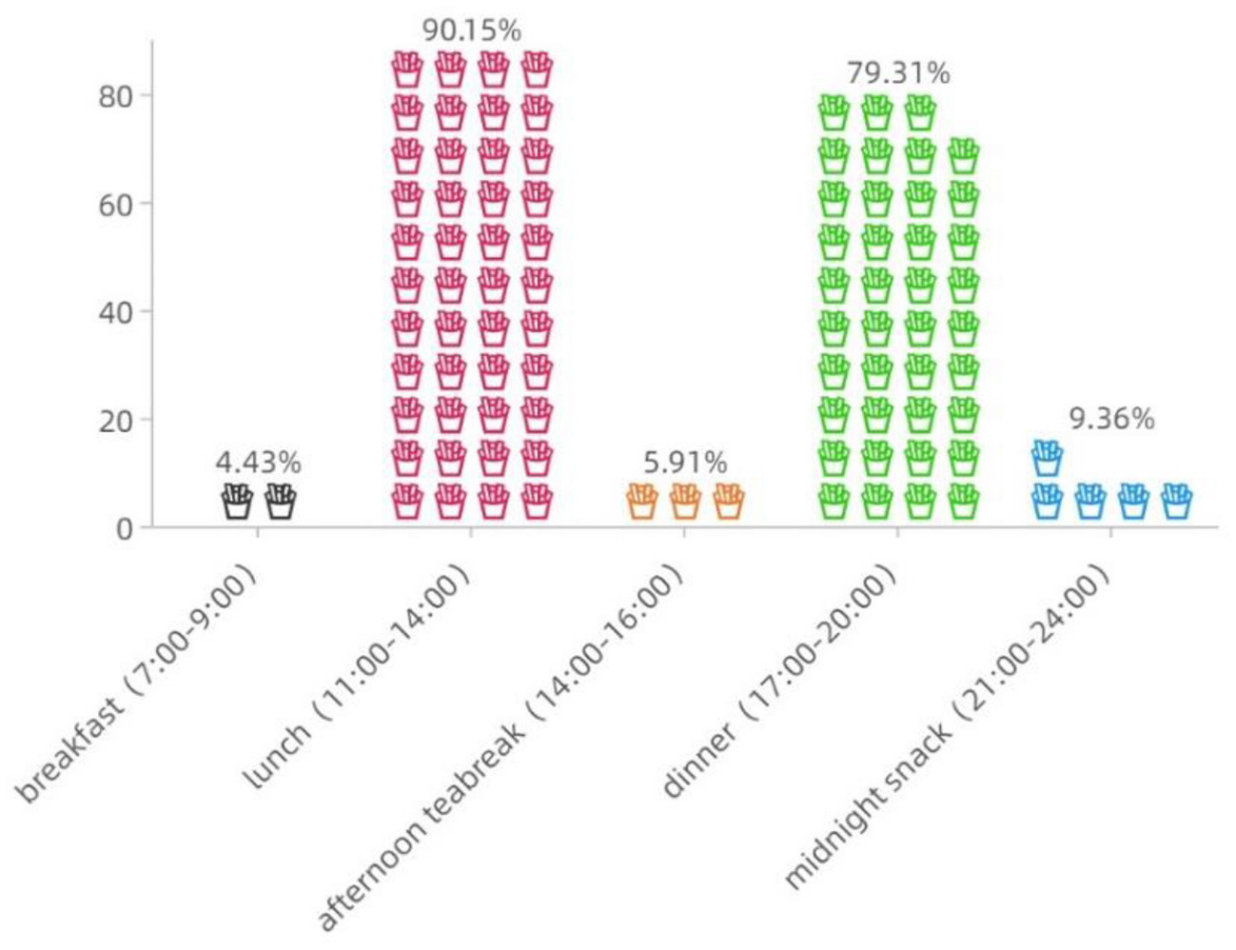
Peak delivery hours for deliveryman during a day.

According to survey data from both questionnaires, the daily peak hours of OFD work and food ordering are highly coincident, which occurring at two time periods (i.e., 11:00–14:00, and 17:00–20:00) (see Table 2 and Figure 1). The mode values of daily OFD orders, and delivery distance for each order are 30–40 (accounting for 40.39%), and 2–4 km (accounting for 70.44%), respectively. This means that deliverymen strive to accomplish the large amount of delivery tasks within a short period of time.

**Table 2:** Distribution of OFD order time intervals from customers.

**OFD order time slots**	**Early morning breakfast (7:00–9:00 a.m.)**	**Lunch (11:00 a.m.−1:30 p.m.)**	**Afternoon snacks (2:00 p.m.−4:00 p.m.)**	**Dinner (5:00 p.m.−8:00 p.m.)**	**late-night supper (9:00 p.m.−0:00 a.m.)**
Percentage %	2.77%	71.23%	9.41%	47.58%	5.39%

After in-depth interviews with deliverymen, actual delivery time has been found to be insufficient to accomplish the tasks for 3 main reasons. First, the default delivery time set by various platforms and merchants is about 30–40 min, which including the durations from origination to merchants, waiting times at merchants, and duration from merchants to destination. The actual time for delivery food from restaurant to customers is usually only 20–30 min or even less. The deliverymen generally deemed that it is difficult to ensure on-time deliveries without violating traffic laws. Second, during peak food demand hours, food production time is usually longer, which leading to extended waiting times at restaurants. Third, traffic congestion, confusing delivery address, unfamiliarity with destinations, and bad weather all contribute to delivery difficulties. All these 3 reasons increased deliverymen's willingness of traffic violations.

**Figure 2:**
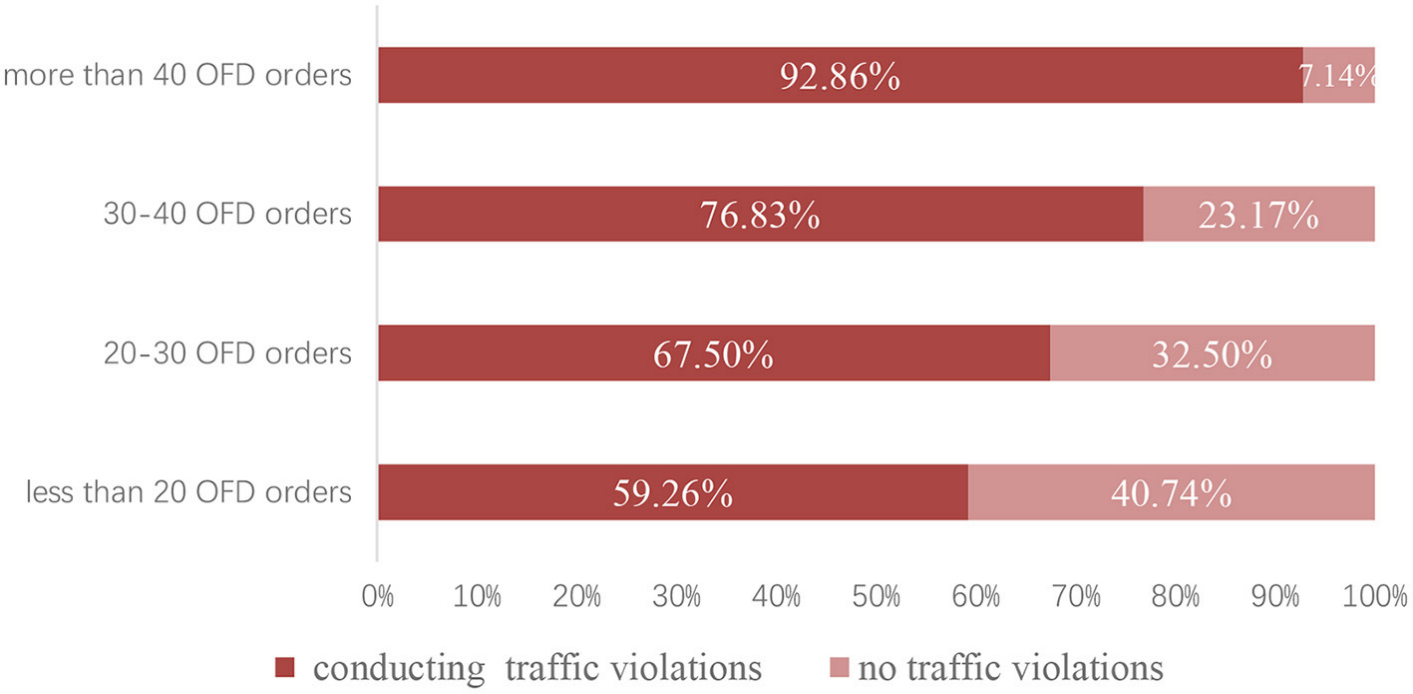
Relationship between the number of OFD orders and conducting traffic violations probability.

Besides, deliverymen usually need to finish a large number of delivery orders assigned by the platform in order to maintain basic income and try to earn more salary. Figure 2 shows that when daily delivery numbers are higher, the proportion of deliverymen conducting traffic violations is greater. This indicates a strong relationship between daily delivery numbers and the traffic violation behaviors.

Therefore, the root cause of traffic violations of deliverymen is the conflict between insufficient delivery time and a large delivery orders to complete within a short period of time since the delivery peaks are pretty fixed.

Although deliverymen strive to meet the on-time OFD demand, data analysis from Questionnaire # 1 results show that the satisfaction rate of consumers for the on-time delivery is >80% (including satisfactory 55.74% and very satisfactory 20.06%). Among the reasons why the deliverymen received bad reviews, over-delayed delivery is the first reason, which accounts for 40.78%. In addition, clicking delivery ahead of schedule (accounting for 14.08%) is also related to over-delayed delivery, which is a violation operation taken by the deliverymen when the over-delayed delivery is upcoming. The survey data shows that bad reviews and complaints are common among customers who are not satisfied with the OFD service. Indeed, 52% of surveyed deliveryman received bad reviews in the last 3 months. Deliverymen pay penalties of about 50–100 yuan for each bad review, while they only receive a reward of 5–10 yuan for each delivery order. In some cases, the penalty for complaint can be even higher at 200 or even up to 500 yuan. Therefore, to improve the customer satisfaction rate and avoid bad reviews and fines, deliverymen tend to be much more willing to violate traffic rules to save some delivery time, which greatly puts other road users and themselves in danger.

To sum up, the combination highly-concentrated OFD orders and insufficient delivery time are the root cause for traffic violations. To radically cure traffic violations, solutions must be developed with consideration of the root cause.

## Case study investigation

### Concept for hierarchical online food delivery mechanism design

Based on the above analysis, this study proposes a new mechanism titled hierarchical OFD mechanism (abbreviated as HOFDM) from the perspective of reducing traffic violations and safeguarding the safety of deliverymen. This is achieved through by incorporating the opinions of deliverymen obtained from the in-depth interviews. As mentioned previously, the root causes of deliveryman conducting traffic violations are highly-concentrated OFD orders and insufficient delivery time. To eradicate traffic violations, traditional safety countermeasures (i.e., industry supervision, penalties) may not work effectively without addressing the root cause.

**Figure 3:**
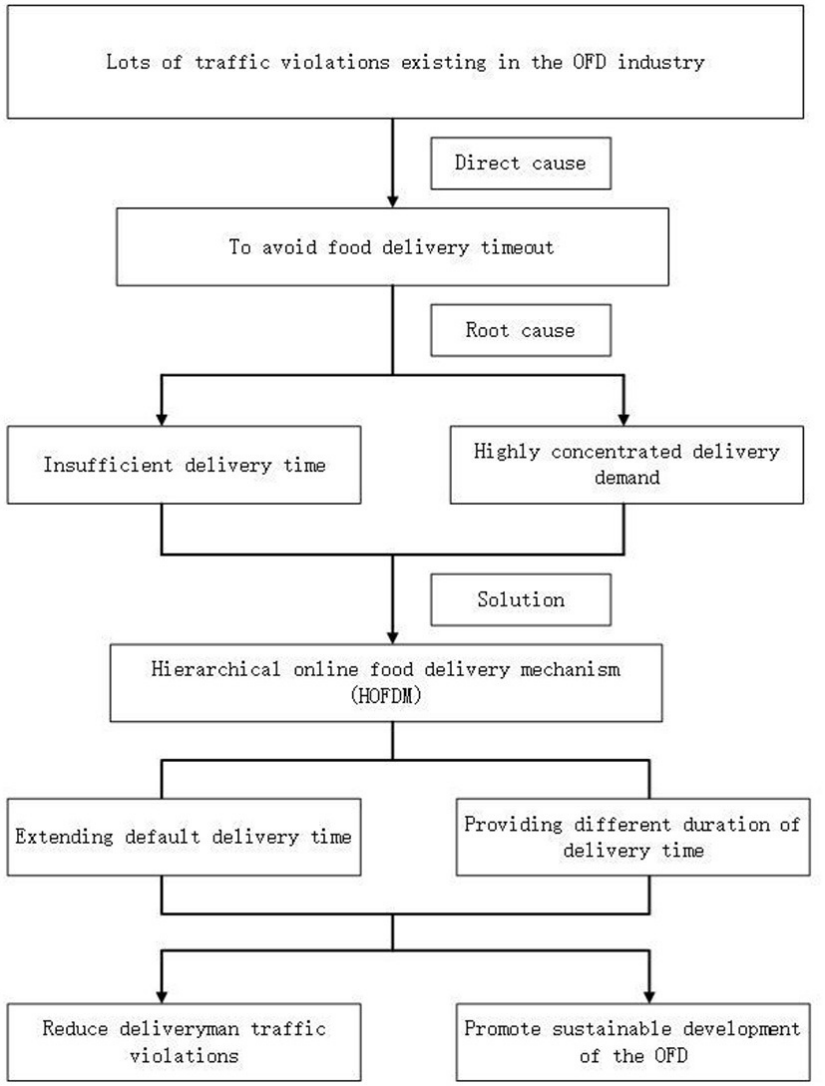
Conceptual framework of hierarchical online food delivery mechanism.

The first issue to deal with is the dispersion of highly-concentrated OFD orders from the customer side. Current delivery systems work with a first-come-first service mechanism. During peak food-demanding hours, the overwhelming large number of OFD orders will often disrupt the system. Thus, customers can be re-directed to make orders outside peak food-demanding hours, thus relieving delivery pressure for deliveryman during those times. Secondly, the default delivery time is setup by the internal algorithm within each platform operated by the merchant. However, deliveries are not configured with any priorities falling under different customer classifications. Therefore, this study proposes the HOFDM to shunt the delivery demand, increase the delivery time, and finally promoting safe deliveries with less traffic violations and working pressure. In such a new system, the delivery service is configured with levels of delivery time and service charges, thus, customers can choose to pay different fees to meet their service needs. This is different from the existing management strategies that cause traffic violations by deliverymen, since HOFDM is designed from the perspective of OFD demands, and sets different levels of delivery according to different flexibility of consumer's timing concerns. Consumers can choose an appropriate service level according to the urgency of their own needs, so that the delivery demand is automatically and effectively shunted. This will leave more delivery time, which not only ensures consumers are satisfied, but also reduces the pressure of deliverymen (see Figure 3 for a conceptual framework and logic of the HOFDM).

Compared with existing OFD mechanisms, the HOFDM approach can provide more diversified delivery services, and achieve the purpose of solving traffic violation problems by changing delivery time and cost. The following key elements need to be addressed for the HOFDM design:

(1) Parameters of the service level. There must be a distinct time and cost for different service levels, so that customers can clearly recognize which level is suitable for them. Meanwhile, such a configuration should be operable and easily accessed through the platforms and by the deliverymen;

(2) Reasonable configuration of service time for each service level. This means that such a configuration should allow sufficient delivery time for deliverymen to reduce their willingness to violate traffic laws, while also taking into account the customers' requirements for fast OFD services;

(3) Additional service charges should be setup according to rationality to enable effective shunting of delivery demand. If charges are too high, then most customers will choose free or low-cost delivery services; otherwise if it's too low, then the majority of customers will choose high priority delivery services. In either case, the demand level cannot be effectively diverted, and the purpose of HOFDM design would not be realized.

Thus, this study further analyzed the configuration of delivery time and service charges based on the survey results and information from in-depth interviews.

### HOFDM configurations for service levels and delivery times

Table 3 shows the acceptance level of customers for durations beyond the default delivery time. The average acceptable extended duration of delivery was 10.61 min. However, considering that the actual delivery time available to deliverymen is much less than that set by the platform, if the standard delivery time is only extended by 10.61 min, it can only make up for part of the delay due to slow meal production speed, traffic congestion and other reasons. Further, there is no guarantee that the delivery person will have sufficient delivery time. If the acceptable extended duration is doubled and added to the existing platform default delivery time, the new standard delivery time for each order is about 50–60 min. Consequently, it is recommended to extend the existing default delivery time limit by 20 min, so that deliverymen have more adequate takeout delivery time, which can effectively reduce their willingness and behaviors of violating traffic rules.

**Table 3:** Consumers' acceptable duration beyond default delivery time for deliveries during workdays and in weekends.

**Duration beyond default delivery time**	**Proportion of consumers**	
	**Workday**	**Weekends**
1–5 mins	19.50%	9.68%
5–10 mins	48.27%	30.29%
10–15 mins	20.33%	28.91%
15–20 mins	9.13%	18.53%
over 20 mins	2.77%	12.59%
Average duration beyond default delivery time (in minutes)	8.97	12.25

Secondly, considering that some consumers have the need to receive food as soon as possible, HOFDM is further proposed with different delivery levels corresponding to different delivery time limits and charging standards for current default and proposed standard delivery time (the difference is 20 min).

Table 4 presents two different HOFDM service levels with 5- and 10-min intervals. It is observed that for HOFDM # 1, the time different among different service levels are too small (only 5 min). Thus, in reality, it is difficult for deliverymen to strictly follow the policy, and it is not easy to attract customers to pay more for higher-level service. Similarly, HOFDMs with >5-min intervals have the same issue and are not explored further in this study. On the other hand, HOFDM # 2 avoids the issues in HOFDM # 1, meaning customers may want to pay more delivery charges corresponding to their urgent service requests. If the interval is <10 min, then in a three level delivery system, it is not operational friendly. Thus, HOFDM # 2 is selected as the final HOFDM in this study based on above discussions.

**Table 4:** Candidate HOFDMs with 5 and 10 min intervals.

**HOFDM 1 with 5 service levels (delivery time in minutes)**		**HOFDM 2 with 3 service levels (delivery time in minutes)**	
Standard delivery service (level 5)	50–60	Standard (Level 3) delivery service	50–60
Level 4 delivery service	45–50	Level 2 delivery service	40–50
Level 3 delivery service	40–45		
Level 2 delivery service	35–40	Level 2 delivery service	30–40
Level 1 delivery service	30–35		

The proposed HOFDM is composed of three service levels including standard delivery (third-level) (i.e., delivery time between 50 and 60 min) without priority delivery fee; second-level delivery (i.e., delivery time between 40 and 50 min) with a certain amount of priority delivery fee; and first-level delivery (i.e., delivery time between 30 and 40 min) with the highest priority delivery fee. Compared to the default delivery time in the current delivery mechanism, default configuration of delivery time for standard and second-level deliveries in proposed HOFDM are extended, thereby leaving more time for deliveryman to finish their tasks. Meanwhile, first-level delivery time in proposed HOFDM is the same as it is in current delivery system. The original intention of HOFDM is to not encourage consumers to choose this level of service. If the number of such service orders is small, and the merchant can cooperate with the proposed HOFDM to prioritize the production of meals that require first-level delivery service, coupled with reasonable order distribution operation, such delivery service requests are expected to be completed within the specified time. The difficulty of delivery tasks will be lessened, and the willingness of deliverymen to violate traffic rules will therefore be reduced.

### HOFDM priority delivery fee analysis based on conditional PSM model

The priority delivery fee plays a key role in order to effectively guide consumers to choose the delivery service level on demand. If the priority delivery fee is too high, the rate of consumers choosing first-level or second-level delivery will be greatly reduced. Although deliverymen receive more delivery time, the purpose of demand diversion is not achieved. When orders are too concentrated, deliverymen still want to take more orders and make more money by violating traffic rules to reduce the delivery time of each order. This does not result in satisfying consumers' online food demand and the development of catering takeout industry, which can lead to difficulty in implementing the mechanism. If the priority delivery fee is too low, consumers will choose first-level or second-level delivery, which will not result in an effective diversion effect, and will not be helpful in solving the traffic violation issue. Therefore, it is necessary to conduct further analysis on priority delivery fees to ensure feasibility of the proposed HOFDM.

The PSM (Price Sensitivity Measurement) model (57) is mainly used to measure the satisfaction and acceptance of target users for different prices, and to determine the price of the appropriate products for users so as to obtain an acceptable range of product prices. The pricing of PSM is carried out from the perspective of consumers' acceptance, which not only considers the subjective will of consumers, but also takes into account the needs of enterprises to pursue the profit maximization. The price testing process is based entirely on the natural reaction to the purchase object, and it is does not involve any competitor's information. However, the PSM model has the drawback of ignoring the purchasing power of consumers. Even if consumers think that the price is reasonable, they cannot purchase due to limited purchasing power and other factors.

In order to reduce the interference degree for test results due to consumers' purchasing power, this study uses the improved conditional PSM model to analyze the sensitivity of consumers to the priority delivery fee based on statistics and experimental psychology theories. The study also constructs a conditional PSM model for analyzing the priority delivery fee by contrasting the “time difference” which replaces “price difference.” The willingness of consumers in paying for the priority delivery fee in different situations is judged and analyzed by the cross analysis. This is between the time grade of consumers' acceptable delivery delay with the original delivery duration and the grade of the early arrival of delivery priority and the difference of the time comparison. In this regard consumers with a smaller time level of acceptable delivery delays under the original delivery duration are more willing to pay a certain fee for a higher level of priority delivery service. For instance, the consumer questions for the second-level delivery are transformed as follows:

1) For the second-level delivery and time difference of the delivery delay that you accept, which price range do you think is cost-effective? (i.e., cheap)
2) For the second-level delivery and time difference of the delivery delay that you can accept, what price range of priority delivery fee would you consider as so low that you may doubt the quality of service and would not buy it? (i.e., very cheap)
3) For the second-level delivery and time difference of the delivery delay that you can accept, what price range of priority delivery fee do you think you may consider is high, but you may still buy it? (i.e., expensive)
4) For the second-level delivery and time difference of the delivery delay that you can accept, what priority delivery fee do you think you may consider is too high to buy it? (i.e., very expensive)

**Figure 4:**
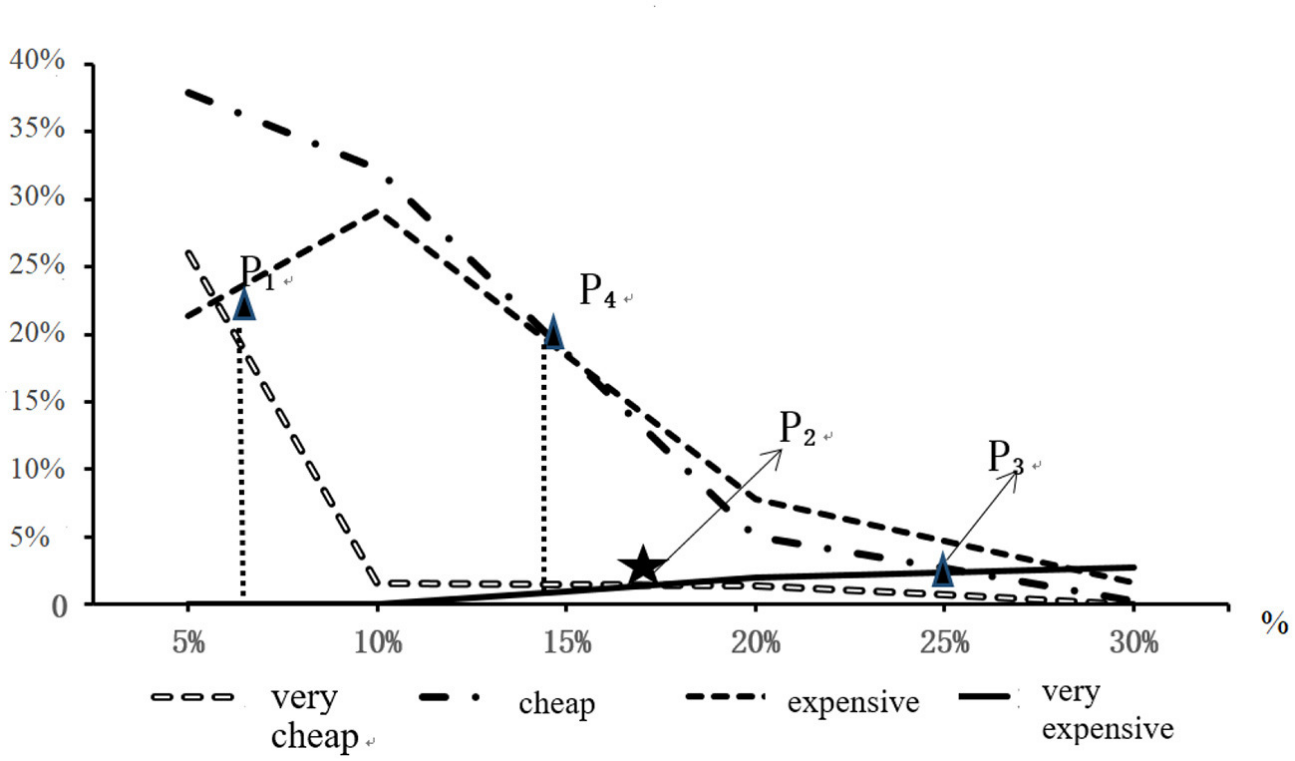
The sensitivity test for the priority delivery fee accounting for the order cost.

By analyzing the payment willingness of consumers in different situations, we summarized the frequency of attitudes of different levels under the percentage that different priority delivery fees accounting for the order cost. Table 5 shows the classification table of attitude toward the order cost under different conditions. Figure 4 is the corresponding graph that shows the highest, the relatively high, the relatively low and the lowest cumulative number of people, with the cumulative number of people being divided according to the percentage of each priority delivery fee accounting for the order cost.

**Table 5:** Consumers' s attitude toward the order cost under different conditions.

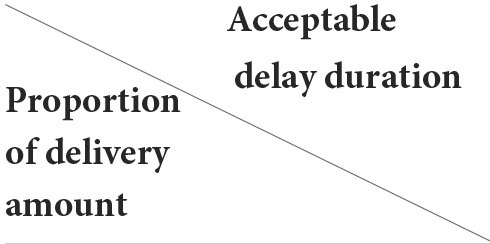		**0–5 mins**		**5–10 mins**		**10–20 mins**		**Over 20 mins**	
							
		**Workday**	**Weekends**	**Workday**	**Weekends**	**Workday**	**Weekends**	**Workday**	**Weekends**
Second-level delivery	0–5%	Very cheap	Very cheap	Cheap	Cheap	Expensive	Expensive	Expensive	Expensive
	5–15%	Very cheap	Cheap	Cheap	Cheap	Expensive	Expensive	Expensive	Expensive
	15–25%	Cheap	Cheap	Expensive	Expensive	Very expensive	Very expensive	Very expensive	Very expensive
	over 25%	Cheap	Cheap	Expensive	Expensive	Very expensive	Very expensive	Very expensive	Very expensive
First-level delivery	0–5%	Very cheap	Very cheap	Very cheap	Very cheap	Cheap	Cheap	Expensive	Expensive
	5–15%	Very cheap	Very cheap	Cheap	Cheap	Cheap	Cheap	Expensive	Expensive
	15–25%	Cheap	Cheap	Cheap	Cheap	Expensive	Expensive	Expensive	Expensive
	0ver 25%	Cheap	Cheap	Expensive	Expensive	Very expensive	Very expensive	Very expensive	Very expensive

The sensitivity test in Figure 4 shows that the scope from point “P1” to point “P3” gives a range of fee proportion acceptable to the consumer. For the fee proportion lower than P1, consumers will not choose priority delivery for doubting about authenticity, while for the fee proportion higher than P3, consumers will also not choose priority delivery because of too high cost. Point “P4” is the acceptable fee proportion, under which the number of people who think that the fee proportion is higher is the same as the number of people who think that the fee proportion is low. Point “P2” is the optimal fee proportion, under which consumers think that the fee proportion is neither too high nor too low. Therefore, the proportion of expenses corresponding to the area where “P2” is located is the optimal fee proportion acceptable to consumers, accounting for about 15–20% of the order cost. Consequently, it can be used as a priority service fee for the second-level delivery. The priority service fee for first-level delivery is supposed to be higher than that for the second-level delivery, but is still within the acceptable range, i.e., it should be on the right side of the range 15–20%, where 20–25% is chosen as the final range for this type of service.

## Results

### Rationality analysis on priority delivery service fee

Based on the questionnaire results and the aforementioned analysis, this part of the case study continues to explore whether the extra monthly cost of priority delivery fee is feasible based on whether such a cost will be a financial burden for potential OFD customers. If it is a feasible cost, the proposed HOFDM can be promoted; otherwise the proposed HOFDM will be difficult to be implemented in reality.

According to the results of Questionnaire # 1, the weighted average value of customers' single takeout order amount is about 25 yuan (proportion of order costs per order per person in detail: 0–20 yuan: 45.92%, 20–30 yuan: 34.02%, 30–50 yuan: 12.17%, 50–100 yuan: 6.23%, more than 100 yuan: 1.66%). Therefore, 25 yuan per order is taken as an example. According to the above-mentioned optimal acceptance fee proportion, it is known that the priority delivery fee per person per order is about 3.75-5 yuan. The second-level priority delivery fee for consumers is about 112.5-150 yuan if the priority delivery fee is used once a day for 30 days (i.e., assumed 30 days per month). As for the first-level priority delivery fee, it is 150–187.5 yuan per month.

Based on the income survey results in Questionnaire # 1, the proportion of the priority distribution fee for different income groups is shown in Table 6. The results highlight that for the majority of respondents, the proportion of second-level priority delivery service fee is concentrated around 1–5%, which is acceptable.

**Table 6:** The proportion of respondents' monthly income and priority delivery service fees.

**Monthly income (Unit: yuan)**	**Less than 1,500**	**1,501–4,000**	**4,001–7,000**	**7,001–10,000**	**Over 10,000**
Proportion of respondents	39.19%	19.45%	21.24%	10.62%	9.49%
Monthly second-level delivery service fees/monthly income	11.25–15%	4.09–5.45%	2.05–2.73%	1.32–1.76%	0.75–1%
Monthly first-level delivery service fees/monthly income	15–18.85%	5.45–6.82%	2.73–3.41%	1.77–2.21%	1–1.25%

1,500 yuan or less will be accounted as 1,000 yuan, 10,000 yuan or more will be accounted as 15,000 yuan, and the rest will be accounted by taking the interval value.

This study compares the daily commuting fees of citizens in Xi'an. According to the current public transport fares, the one-way fare for the unmanned ticket bus is 2 yuan/ticket (i.e., 1 yuan by bus card), and the one-way fare for the subway is 2–6 yuan. The single priority delivery fee is equivalent to the fare of one-way bus or subway. At the same time, according to the relevant literature (58). Table 7 presents the statistical results of the residents' daily commuter expenses survey in 2014. The daily commuting fees of residents range from about 90 to 600 yuan. Most of the respondents' commuting fees range from 140 to 210 yuan, higher than the priority delivery fee. The proportion of the commuting fee is mainly between 3 and 5% of the monthly income. Meanwhile, the values of the consumer price index of traffic fare (Table 8) remained pretty stable in past years according to the Shaanxi statistical year book (59–64). Consequently, the data in Table 7 is meaningful and useful for analysis and comparison.

**Table 7:** The proportion of respondents' monthly income and commuting fees.

**Monthly income (Unit: yuan)**	**Less than 2,000**	**2,000–4,000**	**4,000–6,000**	**6,000–8,000**	**8,000–10,000**	**Over 10,000**
Commuting fees (yuan)	91.8	140.8	205.3	278.6	321.5	571.8
Proportion of respondents	17.5%	54.5%	20.1%	4.8%	1.3%	1.8%
Proportion of commuting fees accounting for monthly income	9.18%	4.69%	4.11%	3.98%	3.57%	3.81%

2,000 yuan or less will be accounted as 1,000 yuan, 10,000 yuan or more will be accounted as 15,000 yuan, and the rest will be accounted by taking the interval value.

**Table 8:** The consumer price index of traffic fare (year 2016–2020).

**Year**	**2016**	**2017**	**2018**	**2019**	**2020**
Consumer price index of traffic fare	101.8	101.2	101.1	99.4	98.8

By comparing the results in Tables 6, 7, the proportion of second-level delivery service fees is almost the same as the proportion of commuting fees, while the proportion of first-level delivery service fees is slightly higher. Therefore, the configurated range of the priority delivery fees in the proposed HOFDM is reasonable.

### Feasibility analysis of the impact of priority distribution fee on flattening peak OFD demand

This part of the study explores the effect of proposed HOFDM on shunting the demand of online food delivery. Firstly, the delivery time under three service levels are distinct from each other, meaning that for those who want to pay less or a smaller amount of priority delivery fees, they must make OFD orders ahead of time compared to what they are doing right now. In this way, deliverymen will receive early notifications of order information for this group of customers. Or for another group of customers, if they make orders at the usual times without paying priority fees, they would receive their orders at a further delayed time compared to current order receiving times. In other words, peak hour order demands will be flattened, thereby satisfying the design purpose of the proposed HOFDM.

Secondly, the shunting effect is further analyzed for different income groups. As can be seen in Table 6, the proportion of priority distribution fees varies significantly with income, which plays an important role in demand diversion. Meanwhile, Table 9 presents the cross-analysis of occupations and income in Questionnaire # 1.

**Table 9:** Occupation-income cross-analysis for online food consumers.

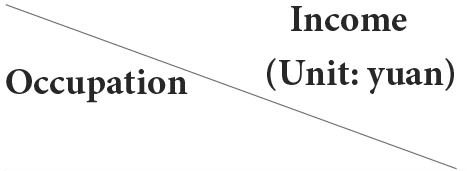	**Less than 1,500**	**1,501–4,000**	**4,001–7,000**	**7,001–10,000**	**Over 10,000**
**(Unit: yuan)**				
Student	94.24%	23.67%	3.10%	2.65%	3.96%
Service industry personnel	0.48%	11.59%	2.65%	3.54%	0.99%
Freelancer	0.48%	3.38%	3.10%	3.54%	1.98%
Worker	0.48%	3.38%	3.10%	1.77%	0.00%
Company employees	0.48%	25.60%	35.84%	35.40%	23.76%
Public institution/ government staff	0.24%	12.08%	25.66%	33.63%	40.59%
Merchant/self-employed personnel	0.48%	2.90%	5.75%	7.96%	19.80%
Retiree/ unemployment personnel	0.72%	4.35%	4.87%	0.88%	0.99%
Others	2.40%	13.04%	15.93%	10.62%	7.92%
Subtotal	416	206	225	113	101

Service industry personnel mainly include: catering staffs/drivers/salesmen/customer service staffs and other industry practitioners; freelancers mainly include: writers/artists/photographers/ tour guides/internet celebrity anchors and other industry practitioners; workers mainly include: factory workers/construction workers/urban sanitation workers and other industry practitioners; and company employees mainly include bank clerks/public relation personnel/human resources and other industry practitioners.

This study takes lunch as an example to conduct the analysis of shunting effect on online food delivery demand because lunch time has the largest OFD demand. The group with an income below 1,500 yuan is mainly students. Although the priority distribution fee accounts for 11.25–15% of their monthly income, students have lots of dining options including school canteens, off-campus small table, home dining and takeout ordering, thereby resulting in lessened dependence on OFD than that of office workers. Besides, their lunch break is much longer than officer workers. Therefore, students are recommended to choose standard (third-level) OFD service or other dining options.

The group with an income between 1,500 and 4,000 yuan is mainly composed of some students, general staff, service industry employees and other personnel, covering multiple society groups. The priority delivery fee is 4.09–5.45% of monthly income. Consumers can choose the delivery level according to their actual demand, e.g., second or standard (third-level) OFD service. The group with income between 4,000 and 7,000 yuan and 7,000 and 10,000 yuan is mainly composed of company employees, public institution/government staffs and other personnel. The priority delivery fee of this group is 2.05–1% and 1.32–1.76% of monthly income. This group has limited lunch time, so takeout ordering is one of the main dining ways. Given the priority, delivery fee accounts for only a minor part of monthly income, and consumers can choose the first and second-level delivery service according to their own demand. The group with an income above 10,000 yuan is mainly composed of company employees, public institution/government staffs, merchants/self-employed personnel and other personnel. The priority delivery fee of this group is merely 0.75–1% of monthly income. The priority delivery fee has little effect on their disposable income, so consumers can consider choosing the first-level delivery service.

It can be observed from the above analysis that the anticipation of the priority delivery fee has an effective shunting effect on distributing demand, thus reducing the delivery pressure of deliverymen, and thereby reducing their willingness to violate traffic rules and the subsequent occurrence of traffic violations. In summary, the feasibility of the proposed HOFDM has been demonstrated and its implementation is viable in Xi'an. For other cities, platforms can formulate the HOFDM according to the local context and situational factors to effectively shunt the level of delivery demand and reduce deliverymen traffic violations.

## Discussion

This empirical study was motivated to reduce the traffic violations by deliverymen from the customer side. According to the strategy of shunting OFD demand and extending delivery time, the propose countermeasures has the capacity to reduce the willingness of deliverymen to violate traffic rules while concomitantly solving the traffic violation problem. However, for the proposed countermeasure to be implemented in real life, this approach requires appropriate attention from multiple entities involved in this system. For example, merchants can optimize their OFD distribution system for improved delivery efficiency, or adopt a salary calculation system by lessening the relationship between salary and the amount of deliveries, or alternatively improve the salary of deliverymen to reduce their willingness for traffic violations. Furthermore, the government can release more incentives for deliverymen to support the OFD system, and especially those from rural areas. Thus, they are more likely to feel protected in this working environment and reduce their willingness for traffic violations.

The results from Questionnaire # 2 indicate that deliverymen often face various pressures, such as those coming from caring for older parents and children (i.e., education), and paying mortgage or rental houses. Therefore, providing affordable housing (i.e., low-rent housing and economically affordable housing), optimizing the enrollment policy for children of migrant workers, and related settlement policies (i.e., those who can settle down in the area after a certain number of years of work can register as a local resident) can all improve their sense of recognition and happiness in a city. In addition, deliverymen will feel welcomed in the industry and will further reduce the willingness to violate traffic rules, and promote sustainable development of the industry. All these countermeasures can also release the pressure and burden of deliverymen, which may enable them work in a better mental health.

## Conclusion and future work

This study investigates and analyzes the root causes of traffic violations that occurred during the OFD process. Based on the analysis and as part of the case study investigation, the study proposed a new hierarchical online food delivery mechanism (HOFDM) with the intend to reduce deliveryman traffic violations. The following conclusions are drawn from the study:

1) The demand of consumers for online delivery is large and the delivery time is highly concentrated during peak food demand hours. The frequent unmet default delivery time is the root factor that leads to frequent traffic violations.
2) The proposed HOFDM has the capacity to reduce traffic violations and the willingness of deliverymen to violate traffic rules by reasonably shunting consumer demand, prolonging the default delivery duration, and providing diversified delivery is better than distribution services.
3) Through applying the sensitivity test of consumers' priority delivery service fee based on the conditional PSM model, we found that the optimal second-level priority delivery fee acceptable to consumers is about 15–20% of the order cost (taking 25 yuan per order for example, the priority delivery fee being about 3.75–5 yuan). The feasibility of HOFDM is further confirmed through the analysis of priority delivery fees.
4) This study suggests that different regions and different platforms can set reasonable division schemes on delivery level and charging standards according to their own conditions, and meet the needs of consumers while reducing deliveryman traffic violations in order to promote the sustainable development of the OFD industry.
5) HOFDM plus other countermeasures can also release the pressure and burden of deliverymen, which may enable them work in a better mental health.

In this paper, the mechanism and feasibility of HOFDM is studied only. In the future, it is expected that further research will be needed to study the details of execution in practice and validate the effectiveness of the proposed HOFDM implemented in different service platforms.

## Data availability statement

The datasets presented in this article are not readily available because the raw/processed data required to reproduce the above findings cannot be shared at this time as the data also forms part of an ongoing study. Requests to access the datasets should be directed to X-wL, lxwvk@chd.edu.cn.

## Ethics statement

Ethical review and approval was not required for the study on human participants in accordance with the local legislation and institutional requirements. Written informed consent from the participants was not required to participate in this study in accordance with the national legislation and the institutional requirements.

## Author contributions

X-wL contributed to the conception of the study, performed data analysis, and wrote the manuscript. X-lG performed data analyses and wrote the manuscript. J-xZ contributed significantly to manuscript preparation. X-bL helped write the manuscript. LL helped revision of the paper. SJ helped draft revision. All authors contributed to the article and approved the submitted version.

## Conflict of interest

The authors declare that the research was conducted in the absence of any commercial or financial relationships that could be construed as a potential conflict of interest.

## Publisher's note

All claims expressed in this article are solely those of the authors and do not necessarily represent those of their affiliated organizations, or those of the publisher, the editors and the reviewers. Any product that may be evaluated in this article, or claim that may be made by its manufacturer, is not guaranteed or endorsed by the publisher.
