# Supplementary material for: Reducing traffic violations in the online food delivery industry—A case study in Xi'an City, China
Source: Front Public Health. 2022 Oct 6;10:974488. doi: 10.3389/fpubh.2022.974488 (PMC9583162; doi:10.3389/fpubh.2022.974488)
Supplement: Supplementary file 1 [file Data_Sheet_1.docx]

**APPENDIX**

**Appendix 1: Introduction of e-scooter used by delivery man in China**

Figure A1 shows the e-scooters used for food delivery in China. Compared to conventional passenger vehicles, e-scooters are much more affordable, and the small size of e-scooters enables deliverymen to take flexible routes in complex and congested city traffic environments.


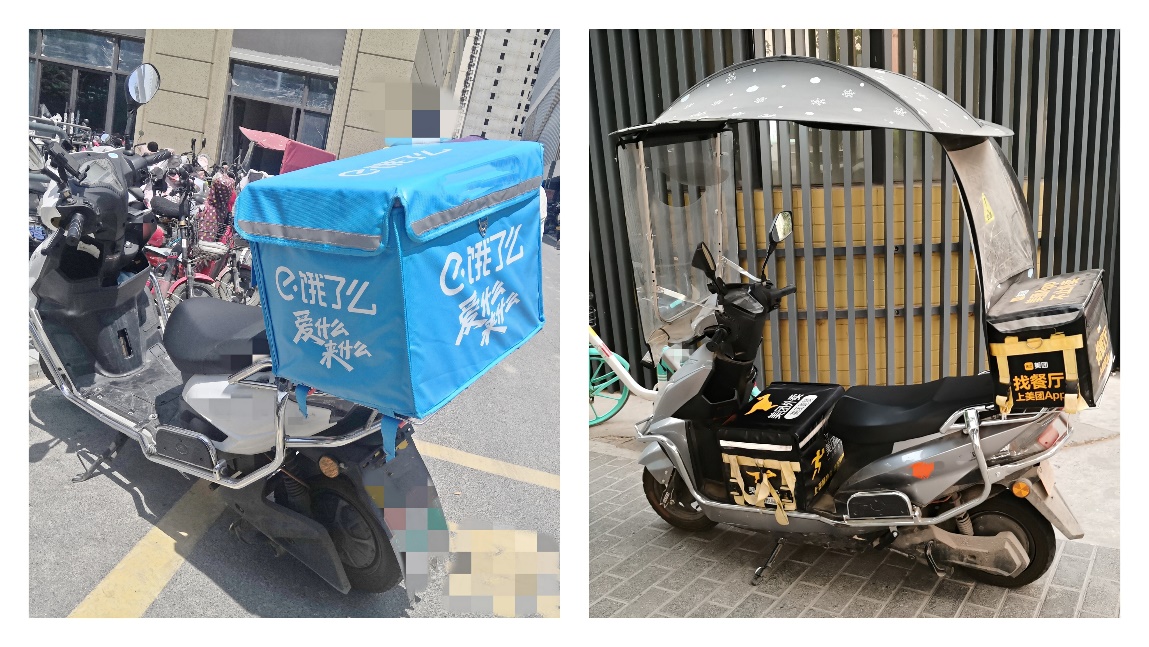


**FIGURE A1**: E-scooters used for food delivery. (taken by authors)

Table A1 compares how e-scooters and convention vehicles adapts to various city traffic environment. Such a comparison indicates that e-scooters are highly like to become the optimal choice for deliverymen in most applications and this includes cities in China.

**TABLE A1**: Comparisons of adaptability to various city traffic environments for e-scooters and convention vehicles (self made by authors ).

| **City traffic environment** | **Conventional vehicles** | **E-scooters** |
| --- | --- | --- |
| Congested road | Stay in the traffic queue with low speed, frequent stops or complete stop. | May take non-roadway lanes (e.g., pedestrian routes) to travel through congested road segments although this is potentially against roadway regulations. |
| One-way lane | Must take the required detours. | May take pedestrian routes to travel instead of detours, although this is potentially against roadway regulations. |
| Parking | Must park in designated parking areas with pay parking fees. If vehicles are parked along road segments with yellow marked edges, they are at risk of receiving fines. | Almost all e-scooters are parked at random places and deliverymen do not always need to pay parking fees. |
| Traffic regulations | Must follow the traffic regulations strictly, otherwise vehicle owners will receive fines and deduction of points from license. | E-scooter driving can be more flexible with less restrictions by traffic regulations. |
